# Molecular-based assay for genotyping *Leishmania* spp. from clinically suspected cutaneous leishmaniasis lesions in the Garmian area, Kurdistan Region of Iraq

**DOI:** 10.1016/j.parepi.2022.e00240

**Published:** 2022-01-24

**Authors:** Hassan Mohammad Tawfeeq, Shahnaz AbdulKader Ali

**Affiliations:** a Nursing Department, Kalar Technical College, Sulaimani Polytechnic University, Sulaimani 46001 Kalar, Kurdistan Region, Iraq; b Department of Microbiology, College of Medicine, University of Sulaimani, Kurdistan Region, Iraq

**Keywords:** Cutaneous leishmaniasis, 18S rRNA, ITS1, Phylogenetic analysis, Kurdistan region of Iraq

## Abstract

Cutaneous leishmaniasis (CL) is highly prevalent in southern Iraq and neighboring countries, but is non-endemic to the Kurdistan Region of Iraq, particularly in the Garmian area. This study aimed to investigate the causative agent of CL at the molecular level by amplifying the small subunit (18S) rRNA and internal transcribed spacer 1 (ITS1) region. The present study was conducted from December 2019 to December 2020 at Kalar General Hospital, Kalar, Kurdistan Region, Iraq. Eighty-five clinical specimens were collected selectively from patients with suspected CL lesions via fine needle aspiration. After parasitic genomic DNA was extracted from the removed fluid, PCR and DNA sequencing targeting the 18S rRNA and ITS1 region were performed for molecular detection and species identification. Additionally, for 14 samples, the target bands of amplified DNA fragments for both 18S rRNA and ITS1 were extracted and sequenced via Sanger method using both the directional primers employed in the PCR. Seventy-one (83.53%) of the 85 suspected patients had CL, based on amplification of 18S rRNA and ITS1 via PCR. The sequence analysis revealed that all samples were *Leishmania major*. Phylogenetic analysis based on ITS1 was also performed. Our study revealed that our molecular method was an efficient technique for detecting CL and a valuable method for identifying *Leishmania* species in clinical samples. Sequence analysis indicated that the causative agent of CL in the Garmian area was *L. major* and the disease was rural in origin.

## 1 Introduction

Leishmaniasis, particularly cutaneous leishmaniasis (CL), is considered as one of the endemic dermal diseases and, as such, is a great public health concern in Mediterranean regions and the Middle East, including Iraq (Ashford et al., 1992; Control and Prevention, 2004). Annual incidence of CL has been reported by the World Health Organization (WHO) as approximately 1.5 million cases per year (WHO, 2010). *Leishmania tropica* and L*. major*, are the main species implicated in CL in Iraq (Al-Warid et al., 2017). Infection is usually transmitted via the bite of various species of infected female sand-flies (Killick-Kendrick, 1999; Burza et al., 2018), although transmission has also been reported as a result of a laboratory accident (Vera-Izaguirre et al., 2006). The disease is locally (Iraq) most commonly known to be spread by *Phlebotomus papatasi and Phlebotomus sergenti*, as two species of a vector. (Salam et al., 2014). Clinical manifestations range from spontaneously healing lesions to chronic and mutilating cutaneous or mucocutaneous ulcers and, rarely, a chronic diffusible cutaneous disease (Dogra et al., 1990).

Laboratory investigation of CL is mainly based on a microscopic examination of Giemsa-stained skin scrapes or fine needle aspirates (De Vries et al., 2015). This method lacks a high sensitivity and specificity, however, and does not provide any clues regarding the species involved in the pathogenesis of the disease (Kumar et al., 2007). Diagnostic techniques of culturing the microorganism require sophisticated laboratory setups, are time-consuming, and carry the risk of cross contamination (Berman, 1997; Bensoussan et al., 2006). Serological tests also possess drawbacks, as they may be complicated by the cross-reaction of antibodies with trypanosomiasis, tuberculosis, and toxoplasmosis (Sundar and Rai, 2002). Another limitation is the variability of host sensitivities as a result of antibody titres that may differ with regard to the causative species, tissue tropism, and the immunocompetence of the host (Alvar et al., 1997). Detection of CL via molecular approaches, specifically polymerase chain reaction (PCR), offers a high sensitivity, making it relevant in chronic cutaneous lesions with lower parasite loads (Eroglu et al., 2014). Currently, a number of PCR techniques are used for diagnostic applications to determine parasites at the genotypic level (Ajaoud et al., 2013). Amplification targets are either genomic (nuclear) DNA—such as the small subunit (18S) rRNA gene (Guillaume et al., 1992), the internal transcribed spacer 1 (ITS1), mini-exon regions, the tubulin gene (Luis et al., 1998), and heat shock protein 70 (Garcia et al., 2004) or extrachromosomal DNA, such as repetitive kinetoplast DNA mini-circles and cytochrome *b* (Belli et al., 1998; Luyo-Acero et al., 2004). Up to now, and as far as is known, whole genome sequencing is still as the most advanced and developed technique for the identification of varies species, as it due to the fact that it is with the highest sensitivity and specificity (Salloum et al., 2020).

To the best of our knowledge, there have been no previous studies involving the detection of CL based on 18S rRNA and ITS1 region carried out in the Garmian area. This study therefore aimed to detect and identify *Leishmania* species in patients with suspected lesions of CL referred to the Kalar General Hospital by using a molecular approach via the detection of 18S rRNA and ITS1, respectively. Sequencing and phylogenetic studies has been conducted to the evolutionary relationship among closely related strain in Iraq and neighbor countries.

## 2 Materials and methods

### 2.1 Study area

**Fig. 1:**
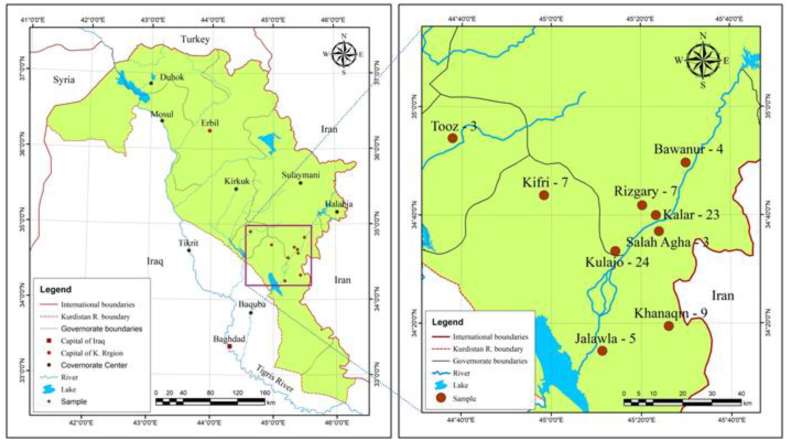
Geographical map of the Kurdistan Region of Iraq and a more detailed view of the Garmian area, showing where study samples originated.

The study was conducted from December 2019 to December 2020 at Kalar General Hospital, Kalar, Kurdistan Region, Iraq. Kalar is the administrative centre of the Garmian area (latitude 34°37′45′′N, longitude 45°19′20′′E), 140 km southeast of Sulaimani and 30 km from the Iranian border. Kalar has a population of approximately 250,000 residents (http://bot.gov.krd/, 2021). Samples came from different regions within the Garmian area (Fig. 1).

### 2.2 Ethical considerations

The study protocol was approved by the Ethical Committee of the College of Medicine, University of Sulaimani (No. 1–15/10/2019). Also, Declaration of Helsinki regarding the ethical principles for medical research involving human subjects was considered by obtaining verbal and written consent from each participant and the guardians of patients under 18 years old before enrolling to the current study.

### 2.3 Sample collection and description

Eighty-five clinical specimens were collected selectively from patients who visited the Kalar General Hospital (a 40 bed hospital which receive patients from the urban and countryside of Garmian area) and were clinically suspected by a dermatologist of having CL lesions, or from patients receiving early antileishmanial therapy. Samples from cutaneous lesions were taken by fine needle aspiration, as follows. After cleaning the lesions with cotton soaked in 70% ethyl alcohol and then allowing them to air dry, 0.1 mL sterile normal saline was injected into active borders of the lesions using a 25-gauge insulin needle. The withdrawn fluid was preserved in absolute ethanol, then transported to the research laboratory of the University of Garmian and stored at 4 °C for DNA extraction and further processing (Amro et al., 2012).

### 2.4 DNA extraction

Before performing DNA extraction, clinical samples were washed twice with normal saline by centrifugation to remove ethanol. Genomic DNA was extracted from pellets using the *EasyPure* Genomic DNA Extraction Kit (TransGen Biotech Ltd., Beijing, China), according to the instructions recommended by the manufacturer. DNA concentration and purity was determined by the ratio of optical density at 260 and 280 nm in a NanoDrop spectrophotometer (Thermo Scientific), and samples were then stored at −20 °C until used in PCR amplification.

### 2.5 Primers

Careful primer design is crucial for the success of any DNA amplification experiment. Based on a sequence of 18S rRNA from *Leishmania* provided by the National Center for Biotechnology Information (Accession no. XR_002460813.1), one pair of primers (labelled F-HMT and R-HMT) was designed to amplify 343 bp. Several other primers were used that had been polymerised by Humanizing Genomics (Macrogen; see Table 1).

**Table 1:** Primer sequences, product size and annealing temperature.

Gene	Primers	Primer sequence(5′ – 3′)	Product size (bp)	Ann. Temp.	Reference
18S rRNA	HMT-F	TCGCAACTTCGGTTCGGTGTG	343	56̊C	This study
	HMT-R	CGGTGCTGACACAGGGTAAAC			
ITS1	LITSR	CTGGATCATTTTCCGATG	350	56̊C	(Schönian et al., 2003)
	L5.8S	TGATACCACTTATCGCACTT			

### 2.6 PCR amplification of 18S rRNA and ITS1

Both PCR assays were performed in a 20μL final reaction volume, which consisted of 10 μL 2x*EasyTaq* PCR superMix (TransGene Biotech; contained *EasyTaq* DNA polymerase, dNTPs, and optimised buffer), 3 μL of DNA template, 0.8 μL of each forward (HMT-F) and reverse (HMT-R) primer for amplification of a partial sequence of 18S rRNA, and of LITSR and L5.8S primers for ITS1. The volume of the reaction was completed with the addition of nuclease free water. PCR mixtures were spun down briefly (5–10 s), then placed in a thermal cycler (TCY, Crealcon, NL) and subjected to the following cycling conditions: initial denaturation at 94 °C for 4 min, 35 cycles of denaturation at 94 °C for 30 s, annealing at 56 °C for 30 s, extension at 72 °C for 40 s, and a final extension step at 72 °C for 8 min.

The amplified DNA fragments were visualised via 1.5% agarose gel electrophoresis, using prime save dye (GeneAid) in TBE buffer at 100 V for 60 min at room temperature. Gels were photographed after electrophoresis and amplicon size was determined by comparison with a 100-bp DNA ladder (TransGene Biotech).

### 2.7 DNA sequencing and phylogenetic analysis

To confirm the determination of species, the target bands of amplified DNA fragments for both 18S rRNA and ITS1 of 14 samples were extracted from the gel using the *EasyPure*® Quick Gel Extraction Kit and sequenced via Sanger method with both directional primers employed in the PCR (Macrogen, South Korea). Individual sequences were aligned, justified, and edited manually using the BioEdit version 7.2.5 software program to form consensus sequences that were submitted to GenBank (National Center for Biotechnology Information, Bethesda, MD, USA) to assign accession numbers. The BLAST software (http://www.ncbi.nlm.nih.gov) was used to reconfirm the species identification results in comparison to the published 18S rRNA and ITS1 sequences in GenBank.

For the phylogenetic study, the sequences of ITS1 obtained from L. *major* in the present study were entered into the MEGA X version 10.2.6 software program (Kumar et al., 2018). The ITS1 sequences were manually cut to a uniform length (312 bp) using BioEdit and then underwent phylogenetic analysis to determine the most appropriate sequence evolution model for the given data, treating gaps and missing data with the partial deletion option. The sequences were aligned using CLUSTALW alignment for constructing trees of evolutionary development. The trees of all isolated species were constructed based on the Neighbor-joining (NJ) method and Tamura-Nei model (Tamura and Nei, 1993). Initial trees for the heuristic search were obtained automatically by applying Neighbor-Join and BioNJ algorithms to a matrix of pairwise distances estimated using the Tamura-Nei model.

### 2.8 Data availability

Fourteen (20%) of positive PCR (Gel purified) products from each sample of 18S rRNA and ITS1 were sequenced, and the results were submitted to Genbank under accession numbers MZ520144-MZ520157 and MZ502957-MZ502970, respectively.

## 3 Results

**Fig. 2:**
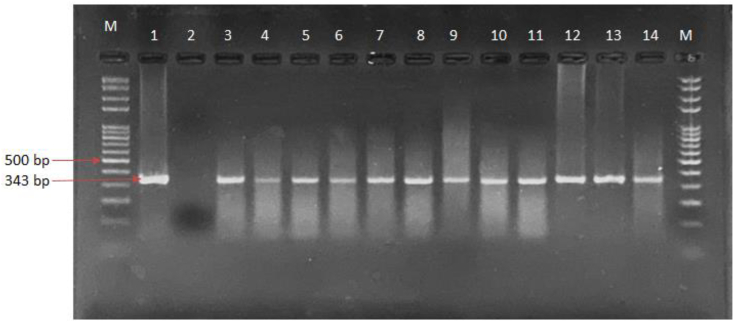
Agarose gel electrophoresis of 18S rRNA PCRproduct of Leishmania isolates. Lane M: 100 bp DNA marker; Lane,1 *L. major* (positive control 343 bp); Lane 2, negative control; Lane 3–14 *L. major* isolates from skin lesions of the patients.

**Fig. 3:**
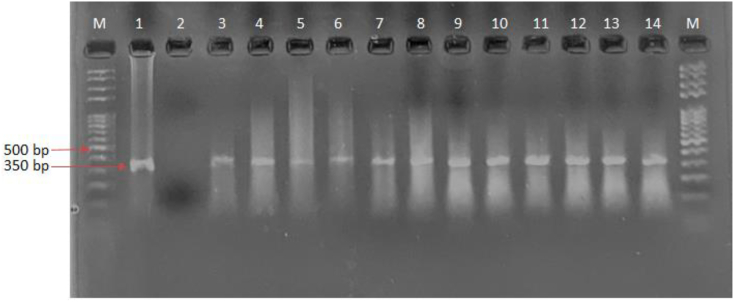
Agarose gel electrophoresis of ITS1 PCR product of Leishmania isolates. Lane M: 100 bp DNA marker; Lane,1 *L. major* (positive control 350 bp); Lane 2, negative control; Lane 3–14 *L. major* isolates from skin lesions of the patients.

A total of 85 patients clinically suspected of CL by a dermatologist were enrolled in this study, 46 (54.12%) of whom were female and 39 (45.88%) male. Patient's ages ranged from one year to 65 years (Average: 30.48 ± 15.36). Although dermatologic problems can be observed during clinical examination, a suspected case of CL should confirmed using laboratory methods. The molecular technique used for detection of CL in this study—PCR amplification of a partial sequence of 18S rRNA from *Leishmania* followed by gel electrophoresis—found that 71 patients (83.53%) were positive for CL, as determined by observation of expected bands 343-bp in length (Fig. 2). In addition, the species of *Leishmania* involved was identified by PCR targeting the leishmanial ITS1 region, with gel electrophoresis showing single bands with a product size of approximately 350 bp (Fig. 3).

**Fig. 4:**
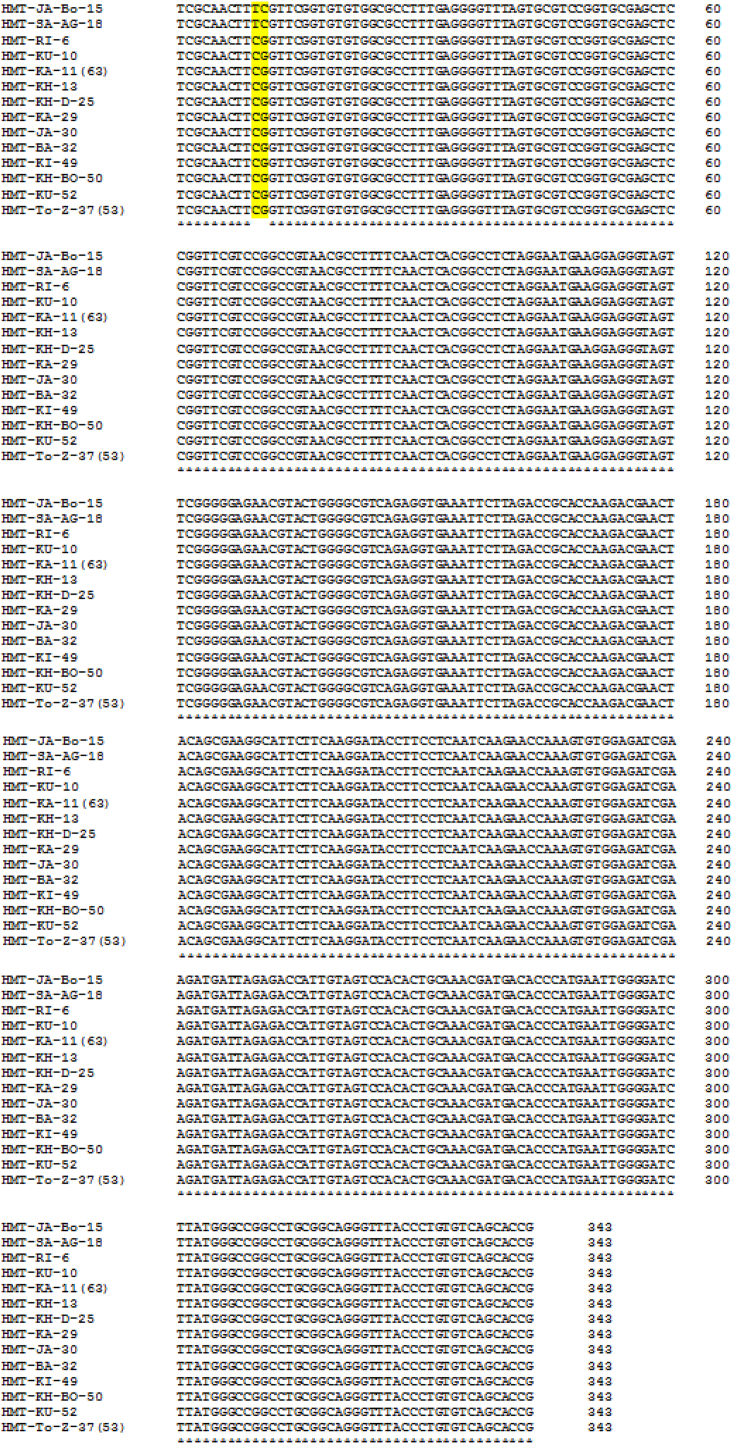
Multiple sequence alignment for 18 s rRNA of fourteen isolates.

**Fig. 5:**
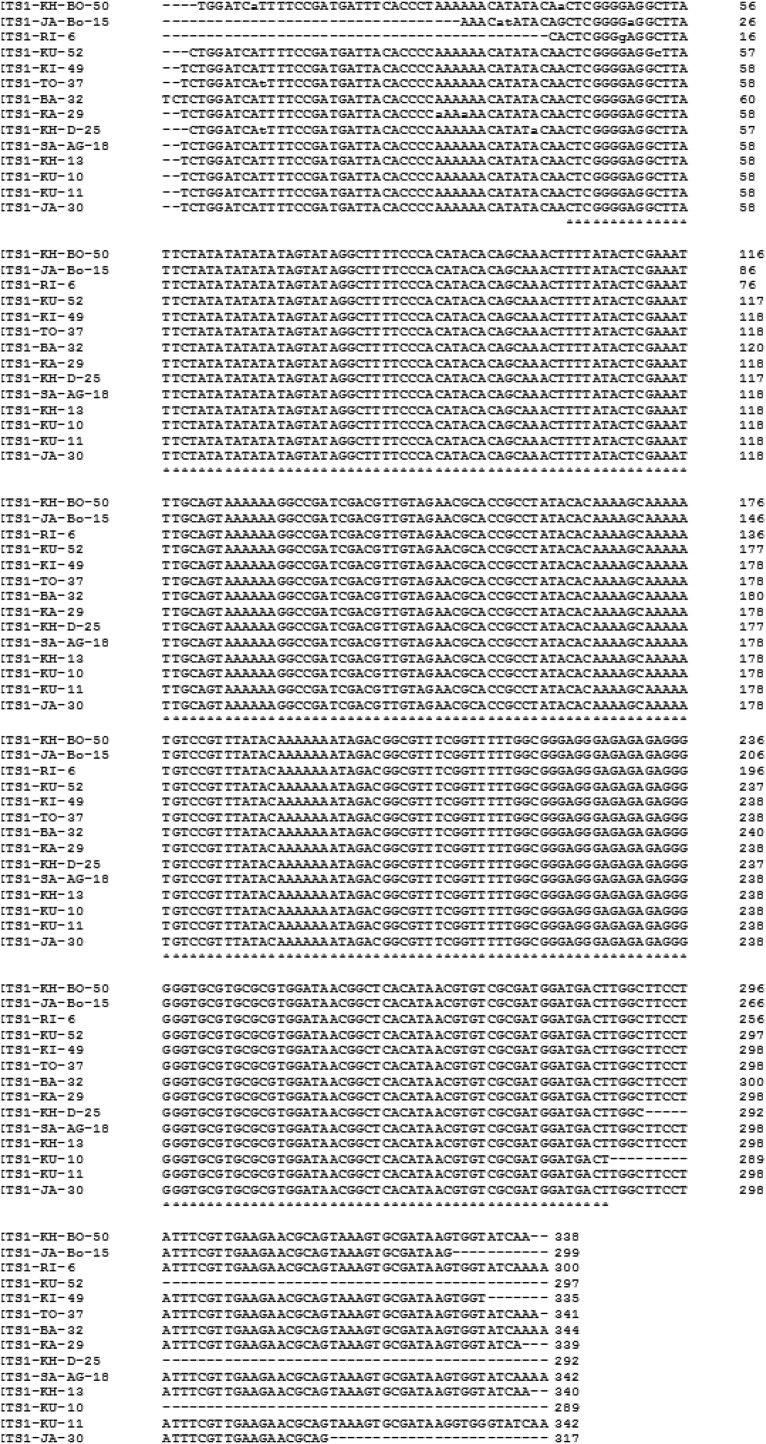
Multiple sequence alignment for ITS1 of fourteen isolates.

To confirm the identification and build phylogenetic trees, 14 (20%) positive PCR products from each sample of 18S rRNA and ITS1 were sequenced and the results were submitted to Genbank under accession numbers MZ520144-MZ520157 and MZ502957-MZ502970, respectively. When the 14 sequences of each 18S rRNA and ITS1 from isolates were aligned, only two nucleotide variations (0.58%) were discovered for 18S rRNA (Fig. 4), at alignment positions 10 and 11 from isolates HMT-JA-Bo-15 and HMT-SA-AG-18, respectively, and no variation was observed for ITS1 (Fig. 5).

**Fig. 6:**
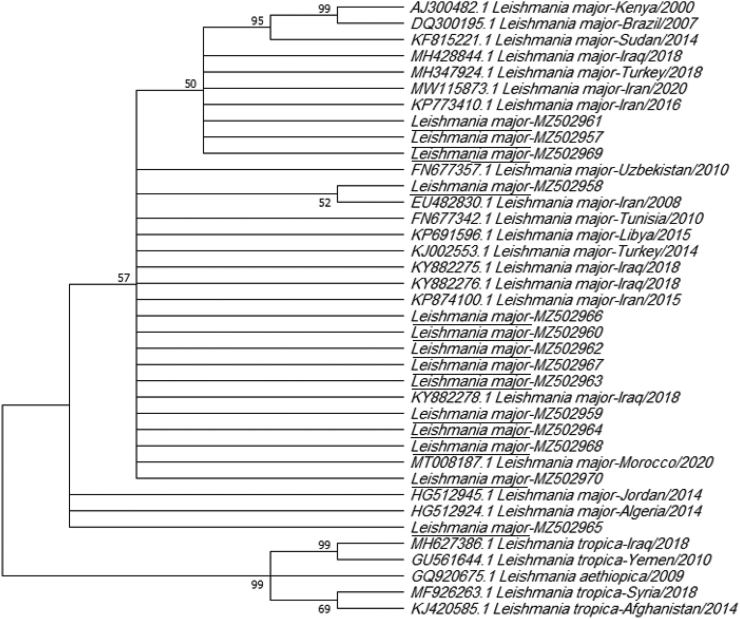
Neighbor-joining (NJ) tree based on the ITS1 sequences. Bootstrap values are based on 1000 replicates. Underlined species represent L. *major* sequences identified in this study.

When the ITS1 sequences obtained in this study were subjected to phylogenetic analysis together with those from representative *Leishmania* strains in the NCBI database (Table 2), the phylogenetic tree showed two main clades, with the L. *major* in the present study located within the same clade as other L. *major* samples from different countries (Table 2). No local isolates fit into a lower clade or formed a different clade with *L. tropica* and *L. aethiopica*. (Fig. 6).

**Table 2:** Nucleotide reference sequences used in this work.

Species	Strain	Origin/host/Year	Accession number
*Leishmania major*	MHOM/IR/07	Iran/human/2008	EU482830.1
*Leishmania major*	isolate 31 mehran	Iran/human/2016	KP773410.1
*Leishmania major*	isolate Lm3–906	Iran/human/2015	KP874100.1
*Leishmania major*	isolate D5	Iran/human/2020	MW115873.1
*Leishmania major*	MHOM/TR/2016/HRURFA123	Turkey/Human/2018	MH347924.1
*Leishmania major*	clone LMJI4	Iraq/Human/2018	KY882278.1
*Leishmania major*	clone LMJI4	Iraq/Human/2018	KY882276.1
*Leishmania major*	clone LMJI4	Iraq/Human/2018	KY882275.1
*Leishmania major*	MHOM/TN/97/LPN162	Tunisia/Human/2010	FN677342
*Leishmania major*	MHOM/IL/81/FRIEDLIN	Brazil/Human/2007	DQ300195.1
*Leishmania major*	isolate Adana	Turkey/Human/2014	KJ002553.1
*Leishmania major*	isolate Z93R	Morocco/Human/2020	MT008187.1
*Leishmania tropica*	strain Kurd3	Iraq/Human/2018	MH627386.1
*Leishmania tropica*	strain 124b	Syria/Human/2018	MF926263.1
*Leishmania tropica*	isolate 64	Afghanistan/Human/2014	KJ420585.1
*Leishmania major*	ITS1	Iraq/Human/2018	MH428844.1
*Leishmania major*	isolate 110 clone 39	Sudan/Human/2014	KF815221.1
*Leishmania major*	isolate Yefren 1	Libya/Human/2015	KP691596.1
*Leishmania tropica*	isolate 10	Yemen/Human/2010	GU561644
*Leishmania major*	MTAT/KE/	Kenya/Human/2000	AJ300482.1
*Leishmania aethiopica*	isolate 1214 clone 2	Ethiopia/Human/2009	GQ920675.1
*Leishmania major*	MHOM/UZ/02/17 h	Uzbekistan/Human/2010	FN677357.1
*Leishmania major*	MHOM/JO/90/JH39	Jordan/2014	HG512945.1
*Leishmania major*	MHOM/DZ/89/LIPA228	Algeria/2014	HG512924.1

The ITS1 gene sequences obtained in this study were subjected to phylogenetic analysis together with those from representative *Leishmania* strains in NCBI database (Table 2) using Molecular Evolutionary Genetic analysis (Mega X) version10.2.6 (Kumar et al., 2018) (Fig. 6). The evolutionary history was inferred by using the Neighbor-joining method and Tamura-Nei model (Tamura and Nei, 1993). The phylogenetic tree showed two main clades the L. *major* in present study located in the same clade with other L. *major* in different countries (Table 2). On the other hand, no local isolates found at lower clade or made a different clade with L. *tropica* and L. *aethiopica*. Initial trees for the heuristic search were obtained automatically by applying Neighbor-Join and BioNJ algorithms to a matrix of pairwise distances estimated using the Tamura-Nei model.

## 4 Discussion

CL is highly prevalent in countries bordering Iraq, particularly Iran, Syria, and Turkey (Ekşi et al., 2017). In general, the disease is not endemic in the KRI (northern Iraq) (Abdulla et al., 2018), particularly in the Garmian area, but massive migratory flows into the KRI from neighbouring countries due to internal conflicts, and immigration of people from mid- and southern Iraq where the disease is endemic, have gradually increased related cases and disease rates over time (Salloum et al., 2016). Developed and sensitive diagnostic methods are therefore required that can detect parasites directly in clinical samples and differentiate all relative species of *Leishmania* accordingly*.* For this purpose, the conventional method is unreliable because it requires the presence of a relatively high number of viable cells and is also unable to distinguish the parasites at a species level. This could pose a problem especially in the chronic phase of CL, where the numbers of parasites in skin lesions are very low. In contrast, DNA-based techniques are considered valuable criteria for differentiating specifically between various species of *Leishmania* (Mirahmadi et al., 2018).

In present study, *Leishmania*'s amastigote in clinical samples of cutaneous *Leishmaniasis* was detected and typed directly by PCR and DNA sequencing of 18S rRNA and ITS1 genes using specific primers. Since it is undoubtedly that many regions of 18S rRNA gene are either completely conserved or less (partially) conserved from long times ago, it has been proved that these regions are supportive for the elucidation of the relatedness between different phylogenies that are less closely related (Baverstock et al., 1989) successfully amplified a partial region of the 18S rRNA gene. This result is consistent with a study by León et al. (2017), in which they reported that the 18S rRNA marker exhibited the best performance in terms of analytical sensitivity and specificity for the detection of *Leishmania* spp. in Colombia.

Based on percentage identities of nucleotides from GenBank, the online BLAST tool revealed strong signals determined for 18S rRNA, and all the *Leishmania* samples were identified as L. *major* with 100% similarity with previously reported reference gene sequences for 18S rRNA (Marcili et al., 2014; Rogers et al., 2011) isolated from humans and deposited in GenBank under accession numbers KF041809.1 and XR_002460809.1, with the exception of two isolates (HMT-JA-Bo-15 HMT-SA-AG-18), which were 99.42% similar to the reference sequences. This slight change in similarity was due to variation in two nucleotides, in alignment positions 10 and 11 of the sequences of the two mentioned strains.

Various studies have demonstrated that ITS1, a region lying between the genes encoding 18S rRNA and 5.8S rRNA, provides genetic markers for the accurate identification of nearly all medically relevant *Leishmania* parasites, due to the fact that this region is highly conserved among species (Ben Abda et al., 2011; Schönian et al., 2001; Dávila and Momen, 2000; Al-Jawabreh et al., 2006). All sequenced ITS1 samples in the present study aligned 100% with a L. *major* isolate from a patient with CL in the Ilam province, in western Iran (GenBank accession number KP773410.1) (Kermanjani et al., 2017).

In this study, we have reported the first application of sequencing 18S rRNA and ITS1 to differentiate between *Leishmania* species causing cutaneous leishmaniasis in the Garmian area, KRI. Results showed that only L. *major* occurs in this non-endemic area and no other species were identified. This may have been the result of the availability of a large number of animals that serve as reservoirs and natural hosts of L. *major*, particularly rodents and stray dogs. In addition, abundant vectors, such as sand flies, are likely key factors influencing the high rate of human infections. Our current findings are in agreement with a recent study conducted in Diyala, Iraq, which reported L. *major* as the main cause of CL (75% of cases) (Al-Ghabban et al., 2020). Similarly, L *. major* was the only pathogen isolated from CL lesions in different areas of Iran (Yadav and Shrestha, 2017; Namazi et al., 2018; Feiz Haddad et al., 2016) In contrast, a study in Ramadi, Iraq, reported that L. *tropica* had a higher incidence than L. *major* (Al-Fahdawi et al., 2018).

Our phylogenetic tree analysis (Fig. 6) showed that L. *major* isolated in the present study and L. *major* previously recorded in different countries such as Iran, Jordan, Turkey, and Algeria were closely related to each other and clustered in the same clade. This relationship was very well supported in the tree (Fig. 6) inferred from the ITS1 sequence analysis, which indicated that L. *major* in this study was a distinct species with high bootstrap values (Fig. 6), likely because there was no nucleotide variation between ITS1 of L*. major* in the current study and that of other mentioned strains.

Approaches utilising DNA are considered valuable and important techniques to give a meaningful and reliable output concerning the taxonomy of *Leishmania* parasites, as they can detect these parasites even if they are present in lower numbers in clinical samples.

## 5 Conclusion

Molecular techniques are reliable and powerful methods for direct detection and identification of *Leishmania* species from clinical samples. Nevertheless, major obstacles, such as cost, expertise, and the need for laboratory facilities, must be overcome before this approach can be implemented. L. *major* was shown to be responsible for 100% of CL in the study area. DNA sequencing and phylogenetic analyses indicated that L. *major* in the current study had less genetic variation when compared with counterparts in neighboring countries, as well as in different countries around the world. We recommend the application of this technique for identification of *Leishmania* species in both vectors and reservoir hosts in future epidemiological studies. Species identification is a crucial role for control strategies and management of the disease. Therefore, a whole genomic sequence and transcriptomics analysis will be conducted in further studies to describe in-depth characterization of the local isolates.

## Declaration of Competing Interest

The authors declare that there is no conflict of interest.
